# Utilization of potato peel as eco‐friendly products: A review

**DOI:** 10.1002/fsn3.691

**Published:** 2018-07-12

**Authors:** Haftom Yemane Gebrechristos, Weihua Chen

**Affiliations:** ^1^ Institute of Food Science and Technology CAAS Beijing China; ^2^ Key Laboratory of Agro‐products Quality and Safety Control in Storage and Transport Process Ministry of Agriculture Beijing China

**Keywords:** eco‐friendly, organic matter, potato peel, potato processing

## Abstract

An eco‐friendly product has been the primary agenda of twenty‐first century of the global scientists. One of the main focuses is by‐product recycling of food processing industries. It has been long time since food industry byproduct converted into energy and value added products. Potato processing is newly emerging food processing factories in developing countries, and potato is the fourth important crop globally. A dramatic food demand increment had shown in the past two decades. This leads to increase the number of food processing industries. Nowadays, food processing industries particularly processed potato manufactures are expanding and generate a huge volume of potato peel. This by‐product causes environmental pollution due to decomposition. However, food byproducts like potato peel have essential organic matter. So this review introduces the potential use of potato peel as food preservative, pharmaceutical ingredient, renewable energy, and animal feed to promote eco‐friendly food industries.

## 1 INTRODUCTION

In food processing industries, environmentally friendly solution for food by‐products is yet to found and is under investigation (Arapoglou, Varzakas, Vlyssides, & Israilides, 2010). On a global basis, potato is the fourth most important food crop following rice, wheat, and corn (Stearns, Petry, & Krause, 1994). Potato popularly known as “the king of vegetables” because it grows in more than 100 countries (Bhajantri, 2008). In developing countries, potato production has increased at an average rate of five percent per year. Currently the total share of developing countries to global potato production rose from 20% to 52%. This is a remarkable achievement, considering the past two decades (FAO, 2008).

Due to increasing urban populations and rising incomes of people living in developing country, the demand for processed food is dramatically increased (Maria & Freire, 2010). Potato Chips are the most common food processed potato product which produce a large amount of potato peel as by‐product (Stearns et al., 1994). Potato peel is a zero value by‐product, which occurs in huge amounts after processing. Depending on the peeling method used, it ranges from 15 to 40% of the first product mass (Sepelev & Galoburda, 2015).

In food processing industries, the most common cause of environmental pollution is associated with organic wastes decomposition. The decomposition occurs when bacterial and other biological forms use the compounds as a source of food (Pailthorp, Filbert, & Richter, 1987). To avoid such problem, use of potato waste as an antioxidant in food systems due to its high phenol content is mandatory (Sepelev & Galoburda, 2015). Phenolic compounds of potato peel synthesized by potato plant for protection from bacteria, fungi, viruses, and insects (Akyol, Riciputi, Capanoglu, Caboni, & Verardo, 2016).

Therefore, potato waste causes much impact on environmental pollution and unwanted cost loss potato processing industries so identifying an integrated, environmentally friendly solution is prompt and essential. Due to multifunctional nature of potato peel, this review carried out to promote eco‐friendly use of potato peel in food processing industries.

## 2 MATERIALS AND METHODS

In this review paper, 45 journals have been reviewed 70% of the journals were direct on potato peel‐related research works and the 30% of them used as indirect advice for the review basically on food waste management in particular on vegetable wastes. Generally recent available literature on potato waste and its organic nature reviewed with a specific aim on industrial applications and narrated as an option for environmentally friendly product in food processing industries.

## 3 UTILIZATION OF POTATO PEEL

### 3.1 Food preservation

Synthetic food preservatives could be used alone or in combination with natural preservatives both synthetic and natural antioxidants been used in food industry; however, application of synthetics preservatives has potential carcinogenic effects but use of natural preservatives alone has a better advantage for human health with low side effect. As a result, attention has being given to vegetable waste with rich source phenols (Sonia, Mini, & Geethalekshmi, 2016; Tiwari et al., 2009). Phenolic compound is found ubiquitously in plants and is of noticeable interest due to their antioxidant and antimicrobial properties (Pezeshk, Ojagh, & Alishahi, 2015).

Food processing industries generate phenolic‐rich vegetable by‐products, and this has been an area of research investigations as a sources of antioxidants and antimicrobial for food preservation (Pezeshk et al., 2015). The entire tissue of fruits and vegetables is rich in bioactive compounds or phenols but the by‐products have higher contents of antioxidant (Sonia et al., 2016). Due to the suspected long‐term negative health effect, use of synthetic antioxidants and antibacterial on food has become a common concern of consumer safety. Therefore, the food industry has enforced to seek natural alternatives food preservative. Potato peel is one of the most important waste products with sufficient amount of phenolic compound so this could be used as a replacement for the current synthetic antioxidant and antimicrobial.

#### 3.1.1 Antioxidant

Antioxidants inhibit oxidation of lipids in foods and consumption of high concentration synthetic antioxidant has carcinogenic effect unlike the natural antioxidants (Thorat et al., 2013). The antioxidant activity of potato peel extracts has strong radical scavenging ability and prevents oxidation reaction in oily foods (Koduvayur Habeebullah, Nielsen, & Jacobsen, 2010).

The dominant phenolic compounds of potato peel extracts are chlorogenic and gallic acids. These are potent sources of natural antioxidants that prevent oxidation of vegetable oil, and this could stabilize soybean oil oxidation reaction through minimizing peroxide, totox, and p‐anisidine indices (Amado, Franco, Sánchez, Zapata, & Vázquez, 2014; Mohdaly, Sarhan, Mahmoud, Ramadan, & Smetanska, 2010). The ability on minimizing oxidation on vegetable oil, potato peel extracts has equal performance with synthetic antioxidants such as butylhydroxyanisole (BHA) and butylhydroxytoluene (BHT). In comparison with mature potato, young potato peel has excellent source of bioactive phytochemicals nature with antioxidant potential (Arun et al., 2015). However, as compared to the application of synthetic antioxidants, potato peel extracts need to apply in higher amount but still looking the advantage of natural antioxidants than the synthetic, it is a promising source of natural antioxidant that could be used as eco‐friendly product on food industries.

#### 3.1.2 Antimicrobial

More than three‐quarter of the world's population has used medicinal plants for treatment of different disease. Herbal plants are important on prevention against highly pathogenic micro‐organisms, and they are safer means of food preservation (Fatoki & Onifade, 2013; Kadhim Hindi & Ghani Chabuck, 2013). Potato peel extracts have antimicrobial compounds against bacterial and fungal organisms. The antimicrobial nature could be due to the presence of flavonoids and terpenes organic compounds (Nostro et al., 2000). Potato peel has bacteriostatic nature with nonmutagenic behavior and safe to use in food processing industries (Amanpour, 2015; Sotillo, Hadley, & Wolf‐Hall, 1998). Therefore, potato peel extract is the future and natural against foodborne pathogenic microbial and the broad spectrum nature of the plant help to discover new chemical classes of antibiotic substances that could serve as food preservative in food processing industries.

### 3.2 Pharmaceutical Ingredient

Pharmaceutical ingredient is a substance used in a finished pharmaceutical product, intended to give pharmacological activity to cure, mitigation, treatment, or prevention of disease (WHO, 2011). Peels of various fruits and vegetables are generally considered as waste product and are normally thrown away. But they have important elements, which could be used for pharmaceutical purpose (Parashar, Sharma, & Garg, 2014). Potato peel has a number of pharmacological interest compounds like glycoalkaloid which could be used as precursory for steroid hormone (Schieber & Aranda, 2009). When we look, the highest amounts of glycoalkaloids are found in potato peel than the flesh part of potato (Chem, 2009). In addition to this, potato powder has a potential of wound healing activity as antiulcerogenic agent (Dudek et al., 2013). Therefore, use of potato peel as pharmaceutical ingredient is natural, nontoxic, and environmentally friendly. So this could be one of the solutions on prevention of the current threat of drug resistance, emerging disease effective treatment and lower the health damaging side effect of synthetic drugs.

#### 3.2.1 Wound management

Wound is a result of disruption of normal structure of skin. To ensure proper healing process, wound tissue need free of revitalized tissue, clear of infection, and moist. Wound dressings should cut dead tissue, exudate, prevent bacterial overgrowth. Various natural topical agents are available which help wound healing process (Keast, Forest‐, & Forest‐lalande, 2004). Potato peel is one of the natural wound healer herbal plants that has ability to produce high tensile strength of wounded skin (Panda, Sonkamble, & Patil, 2011). About 2.5% in ointment form of potato could trigger cutaneous wound healing through improve cells migration and gastric ulceration through protecting gastric mucosa wrinkles (Dudek et al., 2013). Sterile potato peel dressings are better than gauze alone dressing particularly during the healing phase so potato peel dressings could be an alternative for wound dressing in developing countries (Van de Velde, De Buck, Dieltjens, & Aertgeerts, 2011). To use potato peel for wound dressing is readily available, cheap, easy to apply, less painful, and stored easily. Therefore, potato peel can fulfill the ideal dressing painless, nonadherent, nonallergic, nonantigenic, and antiseptic role.

#### 3.2.2 Glycoalkaloids

Glycoalkaloids are naturally found in vegetable crops. Potato glycoalkaloids are known for their toxic nature but the glycoalkaloids found in potato leaves offer natural protection to the plant against pests (Ginzberg, Tokuhisa, & Veilleux, 2009; McCue, 2009). Potato peel has 43% of phenolic acids and 10% of the glycoalkaloids. The highest amounts of glycoalkaloids found in potato peel unlike of the potato flesh (Chem, 2009). Separation of toxic glycoalkaloids from potato peel prior applying in food processing is important to use for further pharmaceutics products. This can be performed using food grade water/ethanol solvents extraction method (Snchez Maldonado, 2014). The upper limit of glycoalkaloid content on food processing should not be exceeded 20 mg per 100g, and the concentrations found in the peel are 3 to 10 times than the flesh (Handling, Issue, & Cantwell, 1996). As a general trend, there is reduction in alkaloids due to its volatile nature. For instance, during potato processing, partial degradation of caffeic acid and glycoalkaloids occurred. However, the dried potato samples have higher steroidal alkaloids such as α‐solanine and α‐chaconine than fresh samples. The air‐drying techniques had the highest steroidal alkaloid contents than freeze and vacuüm oven‐drying (Handling et al., 1996; Snchez Maldonado, 2014). Therefore, potato peel extracts are not only used for food preservation but also could be used as pharmaceutical ingredient through separating glycoalcaliod from potato peel phenolic.

### 3.3 Source of renewable energy

Fossil fuel demand is increasing globally. This creates rapid depletion of the fossils fuel and influence fuel price. As all knows now the main source of environmental degradation is use of fossil fuel which is a global issue. Due to this reason, interest toward use of renewable energy is increasing from time to time (Singh, 2014). A biological procedure for potential retrieval of organic wastes is an anaerobic digestion that used for biogas production (Krus & Lucas, 2014) and (Krus & Lucas, 2014). To cut environmental pollution and economic benefit, food processing industries are focusing on waste reuse. Potato peel as one of the food wastes which has a remarkable potential on production of renewable energy like biogas (Adeyosoye, Adesokan, Afolabi, & Ekeocha, 2010). Therefore, potato peel wastes could give a lot for the worldwide green economy development of recent agenda.

#### 3.3.1 Biogas production

Food wastes for biogas production have high potential. Fruit and vegetable such as potato peel wastes took 55 days for complete digestion to produce biogas in anaerobic condition (Deressa, Libsu, Chavan, Manaye, & Dabassa, 2015; Sedláček, Kubaská, Lehotská, & Bodík, 2010). Biogas plants also give bio‐manure also of energy production and help to solve problems about waste management and providing clean environment (Singh, 2014). Potato peel waste of an industrial is a mesophilic reactor of biogas production. When chemical pretreatments applied on potato peel, the biogas and CH4 yield improved (Krus & Lucas, 2014). Therefore, to decrease natural disasters such as environmental pollution, deforestation, and desertification, use of food waste as biogas for electric generating is prompt and essential.

### 3.4 Animal feed

The cost of livestock feed is increasing due to rising fertilizer costs and extreme weather. So food wastes are an alternative source of feed ingredients. This can cut feed cost and disposal cost and reduce environment pollution (Rivin, Miller, & Matel, 2012). Food wastes have a high nutritional value for livestock feed (Myer & Johnson, 2010). In developing countries, the demand for livestock products is increasing. But feed deficits are the main problem, so unconventional feed resources play an important role. Fruit and vegetable processing industries generate a huge amount of wastes. Such unconventional resources are an excellent source of nutrients for livestock (Wadhwa, Bakshi, & Makkar, 2013). Potato peel is one of the prominent food wastes that could be used as alternative animal feed due to natural sources of energy and fiber with low levels of protein (Chimonyo, 2017). Therefore, food waste materials as residual wastes used as animal feed ingredients and feed additive is essential. For a long period, wastes have also been fed to swine traditionally without processing.

#### 3.4.1 Pig feed

Feeding food waste and garbage to swine is a common practice. Food waste has most often been used as a source of feed for swine unlike other livestock's. Waste matter provision to swine as feed is encourage due to its high disposal costs and feed conversion efficiency of the animal (Westendorf & Myer, 2015). Potato peel is one of the food wastes produced in bulky with high levels of moisture that led to putrefaction in a short period. This will promote their usage as dietary energy and fiber source for pigs (Chimonyo, 2017). Food waste must be heat‐treated before providing to swine cut the risk of animal diseases and cut harmful pathogens to safeguard pig meat consumers (Westendorf & Myer, 2015). Potato waste feeding has antimicrobial activity by reducing coliform bacteria and improved performance of weanling pigs (Jin et al., 2014). Therefore, considering the dual advantage of potato peel used as feed and antimicrobial for pig is important both environmental and financial sustainability of potato processing factories.

## 4 CONCLUSION

To solve the future and current problems of the global environment issue, conversion of food waste into environmentally friendly product through conversion to value added products is mandatory and timely. Therefore, due to its multiple advantages of potato waste can serve as best response for eco‐friendly industrial products. So research focus should gear toward antimicrobial, antioxidant, and other pharmaceutical ingredients from potato processing industries. Attention has given on low‐cost extraction method, and further investigation should also be vested on innovative products from similar by‐products.

## CONFLICT OF INTEREST

The authors declare that we do not have any conflict of interest.

## ETHICAL REVIEW

This study does not involve any human or animal testing.
